# miR-378a-5p and miR-630 induce lens epithelial cell apoptosis in cataract via suppression of E2F3

**DOI:** 10.1590/1414-431X20209608

**Published:** 2020-04-27

**Authors:** Weiwei Gao, Xiaoqing Zhou, Ruihua Lin

**Affiliations:** 1 Department of Ophthalmology, The People's Hospital of Zhaoyuan City, Zhaoyuan, Shandong, China; 2 Department of Ophthalmology, Shanghai Changzheng Hospital, China Naval Medical University, Shanghai, China

**Keywords:** miR-378a-5p, miR-630, E2F3, Apoptosis, Cataract

## Abstract

Cataract, an eye disease that threatens the health of millions of people, brings about severe economic burden for patients and society. MicroRNA (miR)-378a-5p and miR-630 were recognized as essential regulators in multiple cancers. However, the exact functions of miR-378a-5p and miR-630 in cataract are still unclear. The expression of miR-378a-5p, miR-630, and E2F transcription factor 3 (E2F3) in tissues and cells was measured by quantitative real-time polymerase chain reaction. The 3-(4,5-dimethyl-2-thiazolyl)-2,5-diphenyl-2-H-tetrazolium bromide assay was used to evaluate cell viability. Flow cytometry was conducted to analyze cell apoptosis. The interaction between E2F3 and miR-378a-5p or miR-630 was confirmed by dual-luciferase reporter assay. The expression of proteins E2F3, B cell lymphoma (Bcl-2), Bcl-2 associated X (Bax), and cleaved caspase 3 was detected by western blot assay. The expression of miR-378a-5p and miR-630 was up-regulated whereas E2F3 was down-regulated in human cataract lens tissues compared with normal lens tissues. Depletion of miR-378a-5p or miR-630 enhanced proliferation and reduced apoptosis of human lens epithelial cells. Interestingly, up-regulation of E2F3 exhibited the same trend. Next, dual-luciferase reporter assay validated the interaction between E2F3 and miR-378a-5p or miR-630. The rescue experiments further revealed that E2F3 knockdown could recover miR-378a-5p, and miR-630 inhibitor induced promotion of cell proliferation and inhibition of apoptosis in cataract. miR-378a-5p and miR-630 repressed proliferation and induced apoptosis of lens epithelial cells by targeting E2F3 in cataract, representing a prospective alternative therapy for cataract.

## Introduction

Cataract is a common visual impairment in elderly people and the leading cause of blindness globally (1). The projected number of cataract patients will climb to more than 30 million by 2020 according to census data provided by the USA (2). The risk factors of cataract are complicated, such as smoking, hypertension, obesity, diabetes, drug usage, and age (3–5). In the current development of medical therapeutic strategies, cataract surgery remains the most effective treatment due to the recovery of the pupillary reflex and optimization of light transmittance (6). Nevertheless, poor medical care in developing countries impede favorable therapeutic outcomes. Therefore, it is imperative to develop alternative therapies for cataract.

MicroRNAs (miRNAs) are critical modulators that participate in many physiological processes by regulating gene expression through interacting with the 3′ untranslated region (3′UTR) of the messenger RNA (mRNA) and leading to mRNA degradation or translation repression (7,8). Dysregulation of miRNAs is associated with multiple diseases (9–11). For example, miR-378a-5p contributes to cell proliferation and migration in coronary artery disease by targeting CDK1 (12). Up-regulation of miR-378a-5p induces cell apoptosis through repressing ALDH2 expression in alcoholic cardiomyopathy (13). miR-378a-5p was reported to expedite trophoblast cell growth, migration, and invasion via the regulation of Nodal (14). Moreover, increased miR-378a-5p stimulates tumorigenesis of breast cancer by the intervention of mitotic fidelity and promotion of angiogenesis through targeting GABPA (15). miR-378a was reported to regulate oxidative stress in the pathogenesis of cataract (16,17). As a submit of miR-378a, we speculated that miR-378a-5p might also participate in the regulation of cataract.

miR-630 has been identified as a significant regulator of multiple cancers and is implicated in chemo- and radio-resistance of those cancers. For instance, miR-630 functions as a tumor suppressor to alleviate cell survival in cervical cancer and lung cancer by directly targeting YAP1 and CDC7 kinase, respectively (18,19). In addition, miR-630 was reported to regulate cell motility, invasion, and HER-targeting drug resistance in breast cancer via the interaction with IGF1R (20). Similarly, elimination of miR-630 improves radio-resistance of human glioma through targeting CDC14A (21). Previously, Wang et al. (22) reported that miR-630 is up-regulated (4.14-fold) in HLECs with the presence of H_2_O_2_, exerting the conjecture of the involvement of miR-630 in cataractogenesis. Herein, we intended to explore the function of miR-630 in cataract. E2F transcription factors are widely considered to be significant regulators in various pathological processes such as cell cycle, proliferation, apoptosis, and DNA repair (23). As the targets of retinoblastoma (Rb), E2F1-E2F3 were validated as activators whereas E2F4-E2F8 were certified as repressors in transcription (24). For instance, overexpression of E2F3 facilitates cell viability in melanoma via the variation of copy number (25). Moreover, Gong et al. (26) reported that E2F3 is up-regulated and facilitates cell apoptosis in lens epithelial cells, but it is obscure whether E2F3 has a connection with miR-378a-5p or miR-630 in cataract.

In this report, we aimed to clarify the regulatory mechanism between miR-378a-5p or miR-630 and E2F3 in the progression of human lens epithelial cells in cataract. The expression of miR-378a-5p, miR-630, and E2F3 was evaluated by qRT-PCR. Rescue experiments were performed to uncover the effects of miR-378a-5p/miR-630/E2F3 axis on cataract progression.

## Material and methods

### Tissue samples

Cataract patients (n=25, 15 males and 10 females, 50-70 years old) were recruited from The People's Hospital of Zhaoyuan City. They had not received an eye operation and patients with potential for other eye diseases or damage were excluded. Fresh anterior lens capsules were harvested from those cataract patients undergoing phacoemulsification surgery. Meanwhile, 25 normal transparent lens capsules were obtained from the Eye Bank of the People's Hospital of Zhaoyuan City. These lenses were collected by a doctor from postmortem eyes within 8 to 24 h after obtaining written permission from the deceased’s family. All cataract patients signed the informed consent and the protocols were approved by Ethics Committee of The People's Hospital of Zhaoyuan City.

### Cell culture and transfection

Human lens epithelial cells SRA01/04 were purchased from the Cell Resource Center of the Institute of Basic Medical Sciences, Chinese Academy of Medical Sciences (http://cellresource.cn/contact.aspx) (China) and cultured in DMEM medium (Gibco, USA) supplemented with 10% FBS (Gibco) and 0.05% penicillin/streptomycin. miR-378a-5p mimic (miR-378a-5p), miR-630 mimic (miR-630), miR-378a-5p inhibitor (anti-miR-378a-5p), miR-630 inhibitor (anti-miR-630), negative control (miR-NC), and negative control inhibitor (anti-miR-NC) were purchased from RIBOBIO (China). Small interfering RNA (siRNA) targeting E2F3 (si-E2F3), negative control (si-NC), pcDNA E2F3 overexpression vector (pcDNA-E2F3), and pcDNA negative control (pcDNA-NC) were synthesized by Genepharma (China). Cell transfection was performed using Lipofectamine 2000 (Invitrogen, USA).

### Quantitative real time polymerase chain reaction (qRT-PCR)

The tissues and cells were incubated with TRIZOL reagent (Invitrogen) to extract total RNA. The cDNA for miR-378a-5p, miR-630, and E2F3 was synthesized by All-in-One™ First-Strand cDNA Synthesis Kit (FulenGen, China). Next, qRT-PCR was performed using SYBR green (Applied Biosystems, USA). The relative expression levels were analyzed by the 2^-ΔΔCt^ method (27). Glyceraldehyde-3-phosphate dehydrogenase (GAPDH) was applied for normalizing E2F3, and U6 was used as internal reference for miR-378a-5p and miR-630. The primers for miR-378a-5p, miR-630, E2F3, GAPDH, and U6 were: miR-378a-5p (forward, 5′-GCCTCCTGACTCCAGGTCC-3′, reverse, 5′-GTGCAGGGTCCGAGGT-3′); miR-630 (forward, 5′-TTGAGCTGGATTGGCGGGA-3′, reverse, 5′-TTGACGGATGCGGAGGGCT-3′); E2F3 (forward, 5′-CACTTCCACCACCTCCTGTT-3′, reverse, 5′-TGACCGCTTTCTCCTAGCTC-3′); GAPDH (forward, 5′-AGGTCGGTGTGAACGGATTTG-3′, reverse, 5′-GGGGTCGTTGATGGCAACA-3′); U6 (forward, 5′-ACCCTGAGAAATACCCTCACAT-3′, reverse, 5′-GACGACTGAGCCCCTGATG-3′).

### MTT assay

Transfected SRA01/04 cells were plated into 96-well plates and incubated for 24, 48, and 72 h. After incubation, 10 μL MTT (Beyotime, China) was added to the cells for 4 h. Subsequently, 100 μL DMSO (Sangon, China) was used to incubate cells of each well for 2 h. The absorbance value at 490 nm was detected by a spectrophotometer (Huier, China).

### Flow cytometry

Flow cytometry assay was implemented to assess cell apoptosis. In brief, transfected SRA01/04 cells were inoculated into 24-well plates. After incubating for 48 h, the cells were collected and resuspended. The cell resuspension was then stained using Annexin V-fluorescein isothiocyanate (FITC) and propidium iodide (PI) (Vazyme, China) with 5 μL for 20 min. Ultimately, the apoptosis rate was counted by a flow cytometer (BD Biosciences, USA).

### Western blot

Total proteins were obtained from the transfected SRA01/04 cells and analyzed by western blot. Briefly, the proteins were separated through SDS-PAGE and immediately transferred onto polyvinylidene difluoride membranes (Millipore, USA), followed by the blockage of membranes by 5% nonfat milk for 1 h. Next, the membranes were incubated with primary antibodies against E2F3, Bax, Bcl-2, cleaved caspase 3, E-cadherin, N-cadherin, vimentin, and α-SMA (Abcam, USA) and HRP-conjugated secondary antibody (Sangon). Eventually, the immunized signals were determined by the enhanced chemiluminescence (ECL) reagent (Abcam) and ImageLab software version 4.1 (Bio-Rad Laboratories, USA) was applied for image acquisition and densitometry analysis of the blots in this assay according the description of a previous study (28). The intensity of bands was measured as the total volume under the three-dimensional peak, which could be observed in two dimensions using the "Lane Profile" tool to correct the width of the band, accounting for the area under the shaded peak of interest.

### Dual-luciferase reporter assay

The interaction between E2F3 and miR-378a-5p or miR-630 was proven by dual-luciferase reporter assay. In brief, the sequences of 3′UTR of wild-type (WT) E2F3 (with the binding sites for miR-378a-5p and miR-630) were inserted into the pmirGLO vector (Promega, USA) to form WT luciferase reporters of E2F3 3′UTR WT#1 and E2F3 3′UTR WT#2. Meanwhile, the mutant-type (MUT) reporters of E2F3 3′UTR MUT#1 and E2F3 3′UTR MUT#2 were constructed after the binding sites for miR-378a-5p and miR-630 were mutated. They were co-transfected in SRA01/04 cells with anti-miR-378a-5p/anti-miR-630 or anti-miR-NC to construct the dual-luciferase system. Lastly, luciferase activities from cell lysates were measured using a luminometer (Promega GloMax 20/20 Luminometer) after lysing with 1× passive lysis buffer (PLB; Promega). Renilla luciferase activity acted as the internal control of firefly activity, and the ratio of firefly/renilla was considered as the relative luciferase activity.

### Statistical analysis

Data are reported as means±SD. Statistical analysis was performed by SPSS software (IBM, USA) and GraphPad Prism 7 (GraphPad Inc. USA). The correlation between E2F3 and miR-378a-5p or miR-630 was analyzed by Pearson's correlation coefficient. P<0.05 was considered as statistically significant.

## Results

### Overexpression of miR-378a-5p and miR-630 and low expression of E2F3 in cataract

**Figure 1:**
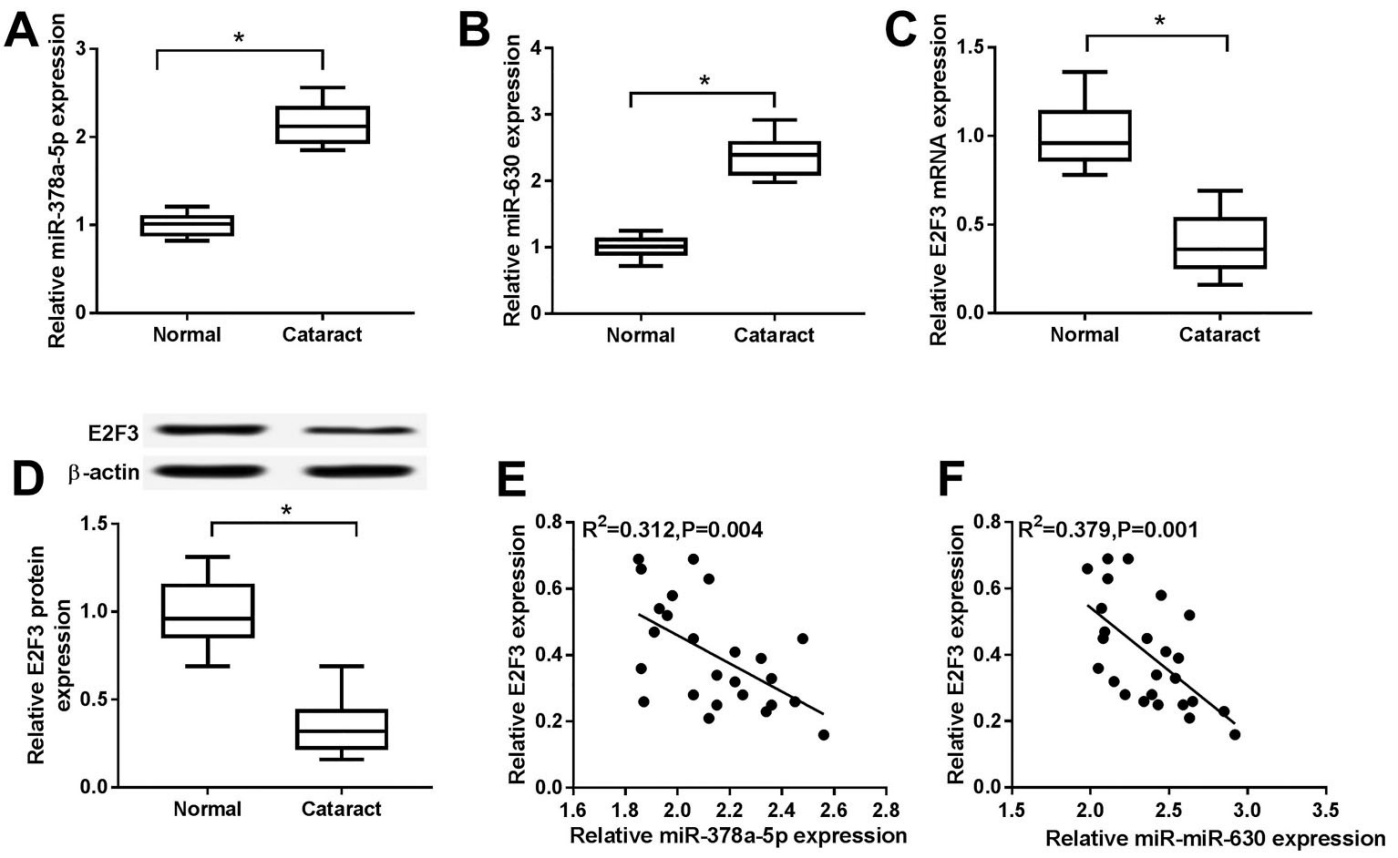
Aand **B**, The expression of miR-378a-5p and miR-630 in human cataract lens tissues or the normal lens tissues was analyzed by qRT-PCR. **C** and **D**, The expression of E2F3 mRNA and protein in human cataract lens tissues and the normal lens tissues was detected by qRT-PCR and western blot. **E** and **F**, The correlation between E2F3 and miR-378a-5p or miR-630 was determined by Pearson's correlation coefficient analysis. Data are reported as median and interquartile range. *P<0.05 (one-way ANOVA followed by Tukey's test).

As illustrated in Figure 1A and B, the expression levels of miR-378a-5p and miR-630 were considerably higher in the anterior lens capsule of cataract patients compared with normal lens tissues. On the contrary, the expression of E2F3 mRNA and protein was remarkably down-regulated in cataract lens tissues compared with the corresponding normal counterparts (Figure 1C and D). More importantly, Pearson's correlation coefficient analysis revealed that E2F3 was negatively correlated with miR-378a-5p and miR-630 (Figure 1E and F). Taken together, miR-378a-5p and miR-630 might expedite the progression of cataract.

### Inhibition of miR-378a-5p and miR-630 facilitated cell proliferation and epithelial-mesenchymal transition (EMT) but repressed cell apoptosis in cataract

**Figure 2:**
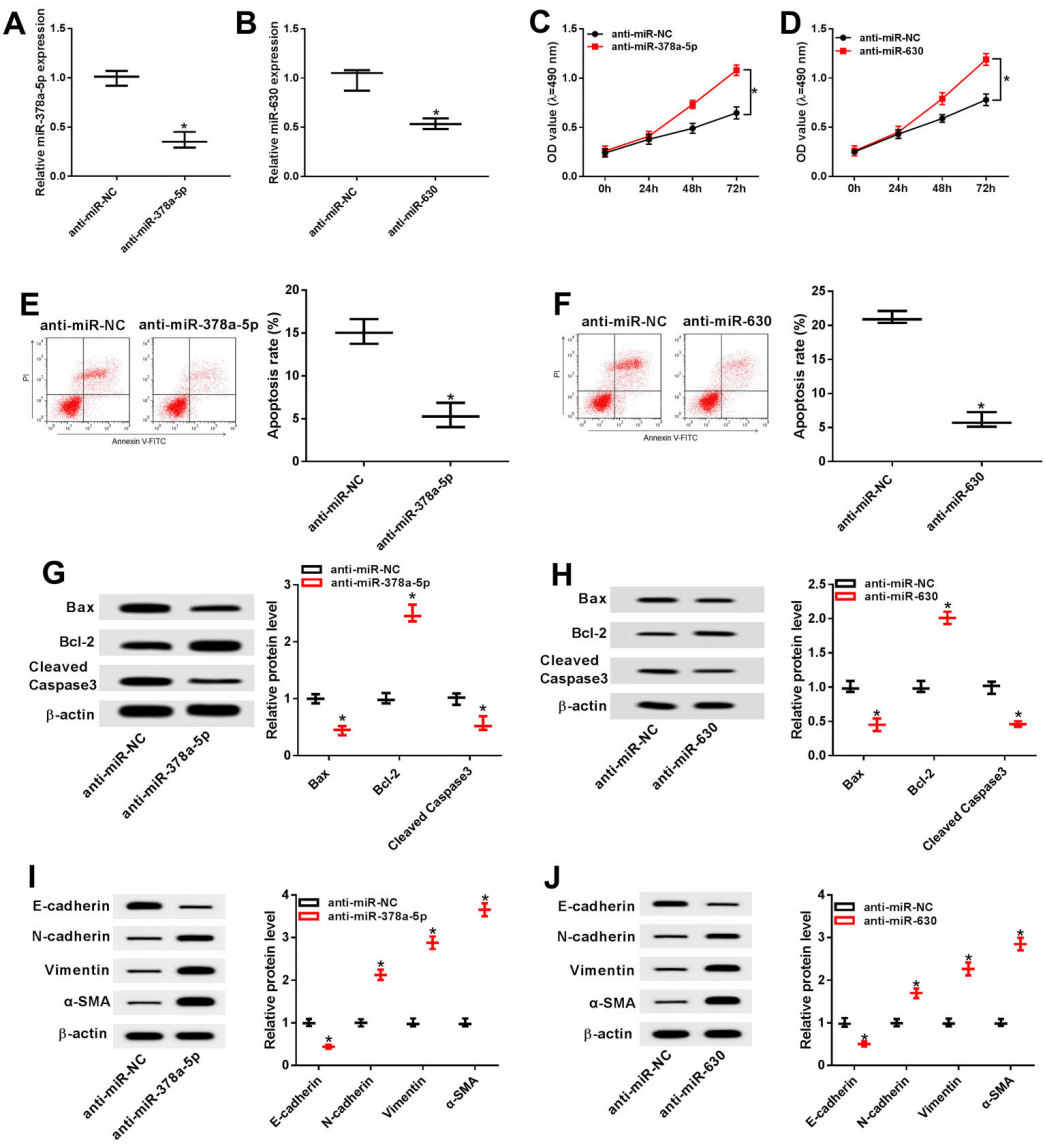
**A** and **B**, The expression of miR-378a-5p and miR-630 in transfected SRA01/04 cells was evaluated by qRT-PCR. **C** and **D**, Cell proliferation was measured by MTT assay. **E** and **F**, Cell apoptosis was analyzed by flow cytometry. **G** and **H**, Protein expression of Bax, Bcl-2, and cleaved caspase 3 in transfected SRA01/04 cells was determined by western blot. **I** and **J**, The protein levels of E-cadherin, N-cadherin, vimentin, and α-SMA were determined by western blot. Data are reported as means±SD. *P<0.05 (*t*-test).

SRA01/04 cells were transfected with anti-miR-NC, anti-miR-378a-5p, and anti-miR-630 to explore the effects of miR-378a-5p and miR-630 on cataract cell proliferation, apoptosis, and EMT. The transfection efficiency was extremely high since the expression of miR-378a-5p and miR-630 was reduced in SRA01/04 cells transfected with miR-378a-5p and miR-630 inhibitors (Figure 2A and B). In addition, down-regulation of miR-378a-5p and miR-630 promoted cell proliferation of SRA01/04 cells (Figure 2C and D). By contrast, cell apoptosis was decreased by miR-378a-5p and miR-630 inhibitors transfection (Figure 2E and F). Western blot results indicated that miR-378a-5p and miR-630 knockdown blocked the generation of pro-apoptosis proteins Bax and cleaved caspase 3 whereas the level of anti-apoptosis protein Bcl-2 was increased (Figure 2G and H). Furthermore, the protein levels of N-cadherin, vimentin, and α-SMA were significantly higher while E-cadherin was notably decreased after down-regulation of miR-378a-5p and miR-630 (Figure 2I and J), implying miR-378a-5p or miR-630 inhibition contributed to the EMT process and the fibrosis. These findings revealed that depletion of miR-378a-5p and miR-630 promoted cell proliferation and EMT, and suppressed apoptosis in cataract.

### Up-regulation of E2F3 promoted proliferation and EMT but attenuated apoptosis of cataract cells

**Figure 3:**
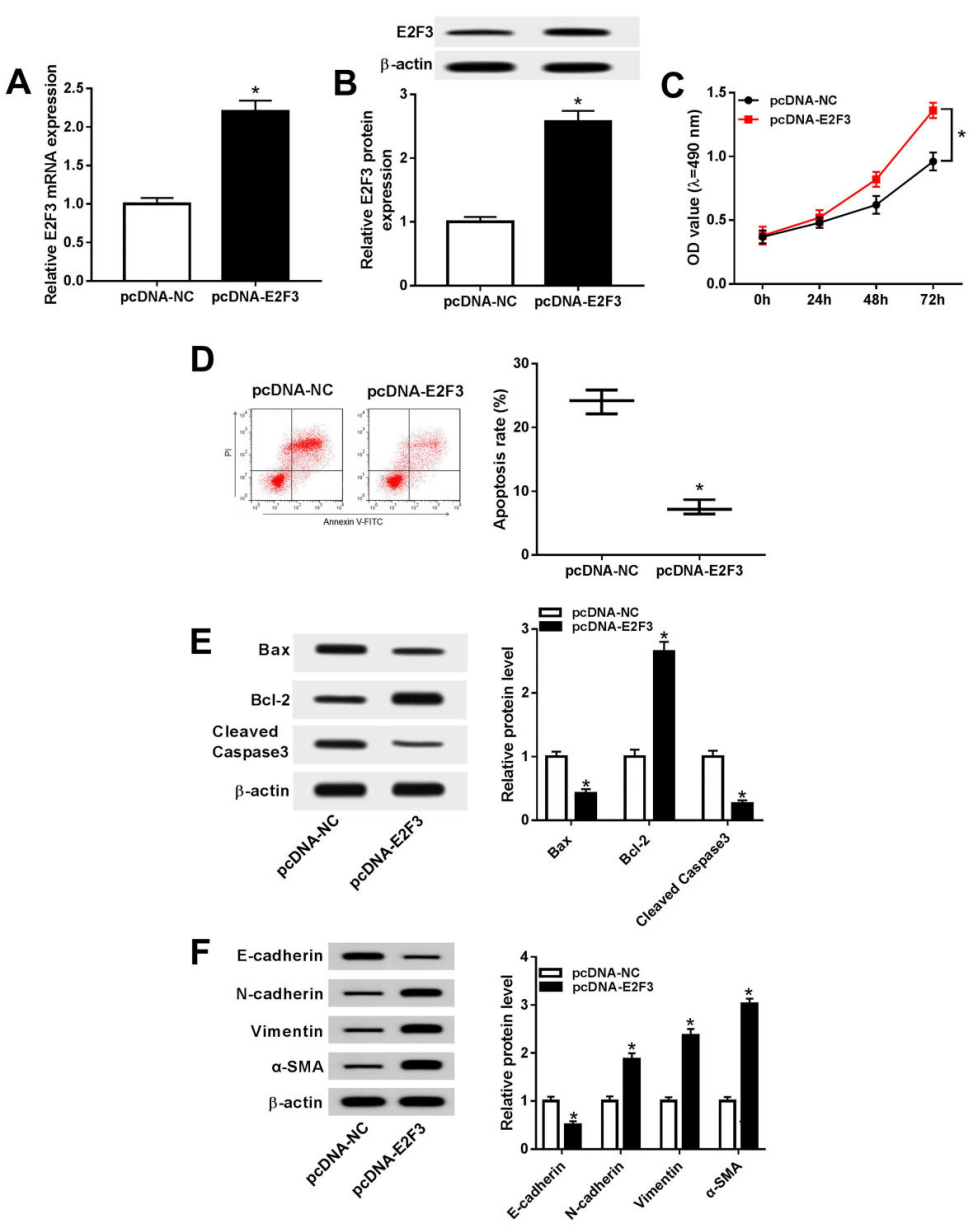
Aand **B,** The expression of E2F3 mRNA and protein in transfected SRA01/04 cells was examined by qRT-PCR and western blot, respectively. **C,** Measurement of cell proliferation was carried out by MTT. **D**, Analysis of cell apoptosis was performed through flow cytometry. **E** and **F**, Protein expression of Bax, Bcl-2, and cleaved caspase 3 (**E**), as well as the protein levels of E-cadherin, N-cadherin, vimentin, and α-SMA (**F**), was assessed by western blot. Data are reported as means±SD. *P<0.05 (*t*-test).

To further illuminate the regulatory effect of E2F3 on cataract cell development, SRA01/04 cells were transfected with pcDNA-NC or pcDNA-E2F3. The expression of E2F3 mRNA and protein was elevated in SRA01/04 cells transfected with pcDNA-E2F3 compared with pcDNA-NC (Figure 3A and B), indicating the success of overexpression of pcDNA-E2F3. Moreover, we discovered that abundance of E2F3 facilitated cell proliferation and restricted apoptosis in cataract (Figure 3C and D). As expected, decreased expression of Bax, cleaved caspase 3 protein and increased expression of Bcl-2 protein was observed in SRA01/04 cells transfected with pcDNA-E2F3 compared with pcDNA-NC (Figure 3E), verifying the inhibition of cell apoptosis by E2F3 again. Regarding EMT, there was a down-regulation of E-cadherin protein but enhancement of N-cadherin, vimentin, and α-SMA proteins in pcDNA-E2F3 group compared with the pcDNA-NC group (Figure 3F), suggesting E2F3 was involved in the advancement of the EMT process of cataract. Therefore, overexpression of E2F3 could improve cell proliferation and the EMT process but alleviate apoptosis in cataract.

### E2F3 acted as a target of miR-378a-5p

**Figure 4:**
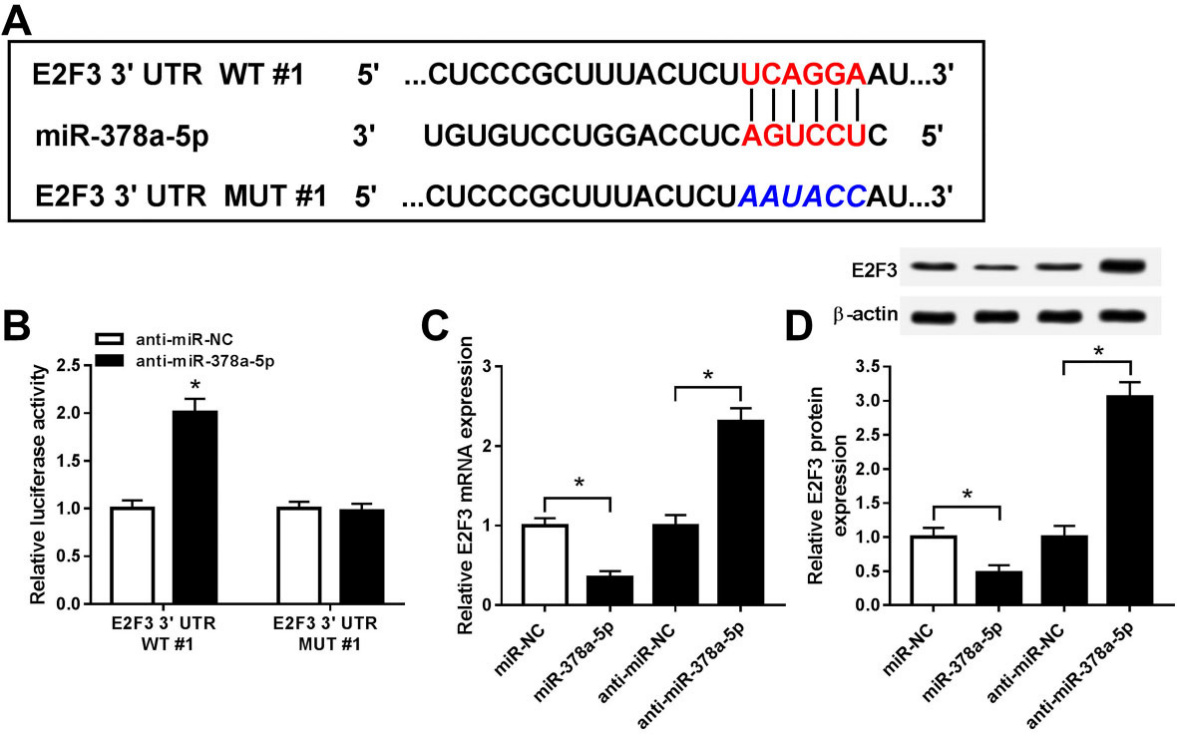
A, The putative binding sites between miR-378a-5p and E2F3 were predicted by Targetscan. **B**, Luciferase activity of SRA01/04 cells co-transfected with E2F3 3′UTR WT#1 or E2F3 3′UTR MUT#1 and anti-miR-378a-5p or anti-miR-NC was measured via the dual-luciferase reporter system. **C** and **D**, The expression of E2F3 mRNA and protein in SRA01/04 cells transfected with miR-NC, miR-378a-5p, anti-miR-NC, and anti-miR-378a-5p was assessed by qRT-PCR and western blot. Data are reported as means±SD. *P<0.05 (*t*-test or ANOVA). WT: wild-type; MUT: mutant-type; NC: negative control.

According to prediction by Targetscan, miR-378a-5p could bind to 3′ untranslated regions (3′UTR) of E2F3 specifically (Figure 4A). To prove the prediction, wild type (E2F3 3′UTR WT#1) and mutant type E2F3 (E2F3 3′UTR MUT#1) luciferase vectors were constructed and co-transfected into SRA01/04 cells with anti-miR-378a-5p or anti-miR-NC, separately. As shown in Figure 4B, luciferase activity was enhanced in SRA01/04 cells co-transfected with E2F3 3′UTR WT#1 and anti-miR-378a-5p, whereas fluorescence intensity remained unchanged in E2F3 3′UTR MUT#1 and the anti-miR-378a-5p transfection group. The expression of E2F3 mRNA and protein was reduced by miR-378a-5p transfection and enhanced by miR-378a-5p inhibitor (Figure 4C and D). All the data demonstrated that E2F3 was a target of miR-378a-5p.

### Interaction between E2F3 and miR-630

**Figure 5:**
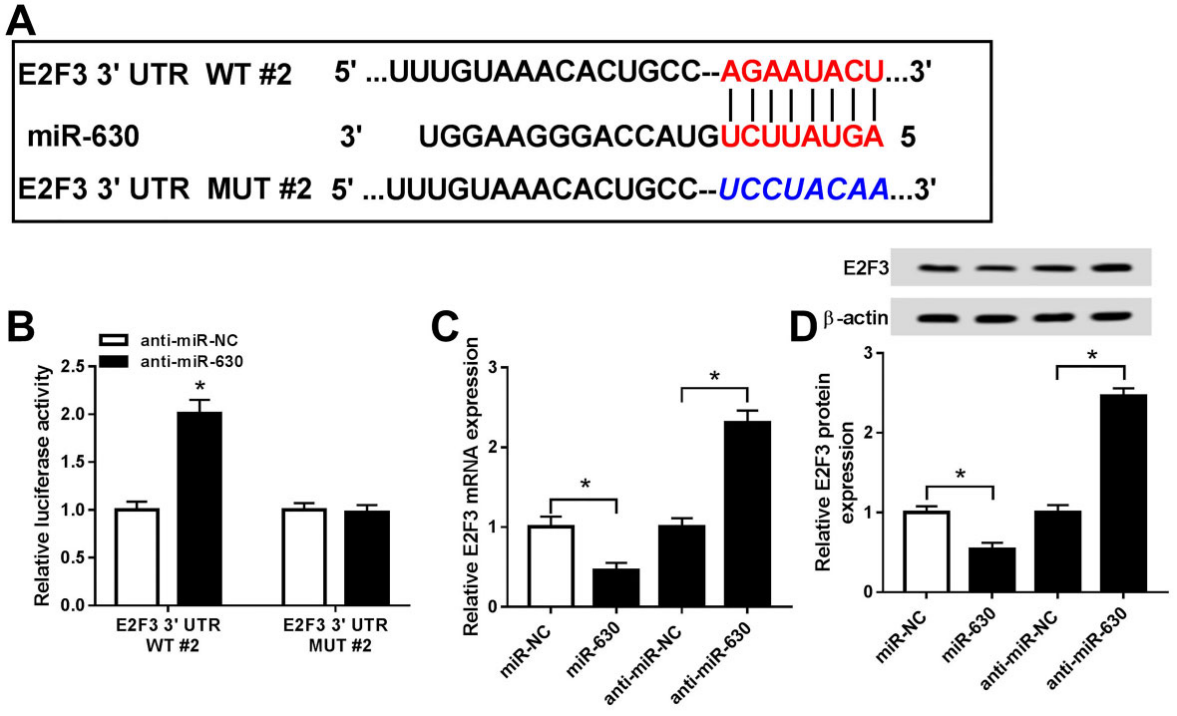
A, The presumptive binding sites between miR-630 and E2F3 were predicted by Targetscan. **B**, Dual-luciferase reporter system was applied for assaying the luciferase activity of SRA01/04 cells co-transfected with E2F3 3′UTR WT#2 or E2F3 3′UTR MUT#2 and anti-miR-630 or anti-miR-NC. **C** and **D**, qRT-PCR and western blot were carried out for detecting the expression of E2F3 mRNA and protein in SRA01/04 cells transfected with miR-NC, miR-630, anti-miR-NC, and anti-miR-630. Data are reported as means±SD. *P<0.05. (*t*-test or ANOVA). WT: wild-type; MUT: mutant-type; NC: negative control.

By searching the online database Targetscan, we noticed that miR-630 contained the binding sites of E2F3 (Figure 5A). The dual-luciferase reporter assay was implemented for validating the interaction between E2F3 and miR-630. The results indicated that miR-630 inhibitor heightened the relative luciferase viability of the E2F3 3′UTR WT#2 group but failed to elevate that of the E2F3 3′UTR MUT#2 group (Figure 5B). Furthermore, the up-regulation of miR-630 suppressed the expression of E2F3 mRNA and protein whereas miR-630 depression exhibited the opposite (Figure 5C and D). Collectively, miR-630 directly interacted with E2F3 in cataract.

### miR-378a-5p and miR-630 regulated the progression of cataract by targeting E2F3

**Figure 6:**
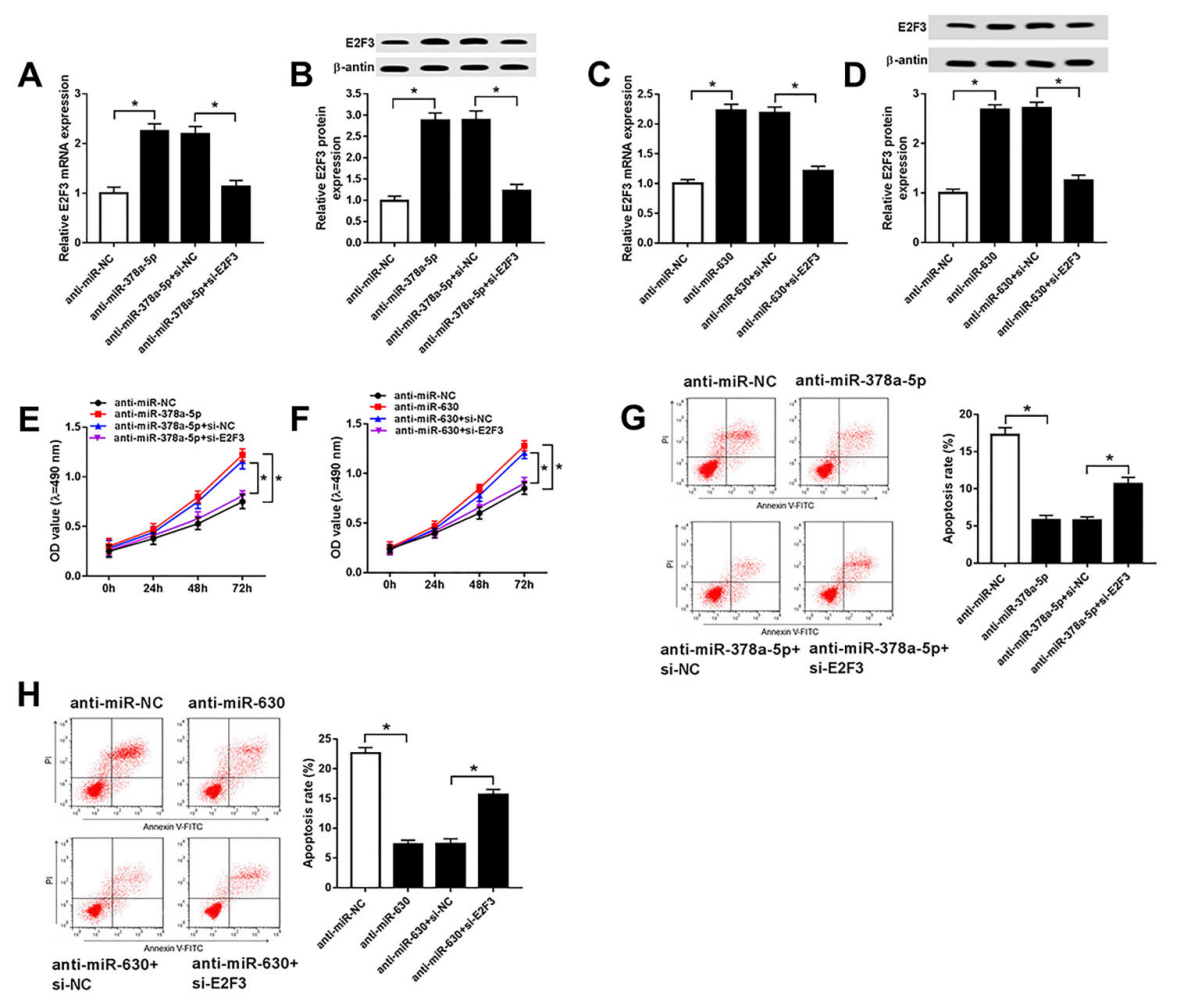
Aand **B**, The expression of E2F3 mRNA and protein in SRA01/04 cells transfected with anti-miR-NC, anti-miR-378a-5p, anti-miR-378a-5p+si-NC, and anti-miR-378a-5p+si-E2F3 was assessed by qRT-PCR and western blot. **C** and **D**, The expression of E2F3 mRNA and protein in SRA01/04 cells transfected with anti-miR-NC, anti-miR-630, anti-miR-630+si-NC, and anti-miR-630+si-E2F3 was measured by qRT-PCR and western blot. **E** and **F**, Detection of cell proliferation in transfected SRA01/04 cells was carried out using MTT assay. **G** and **H**, Analysis of cell apoptosis was conducted by flow cytometry. *P<0.05 (*t*-test or ANOVA).

**Figure 7:**
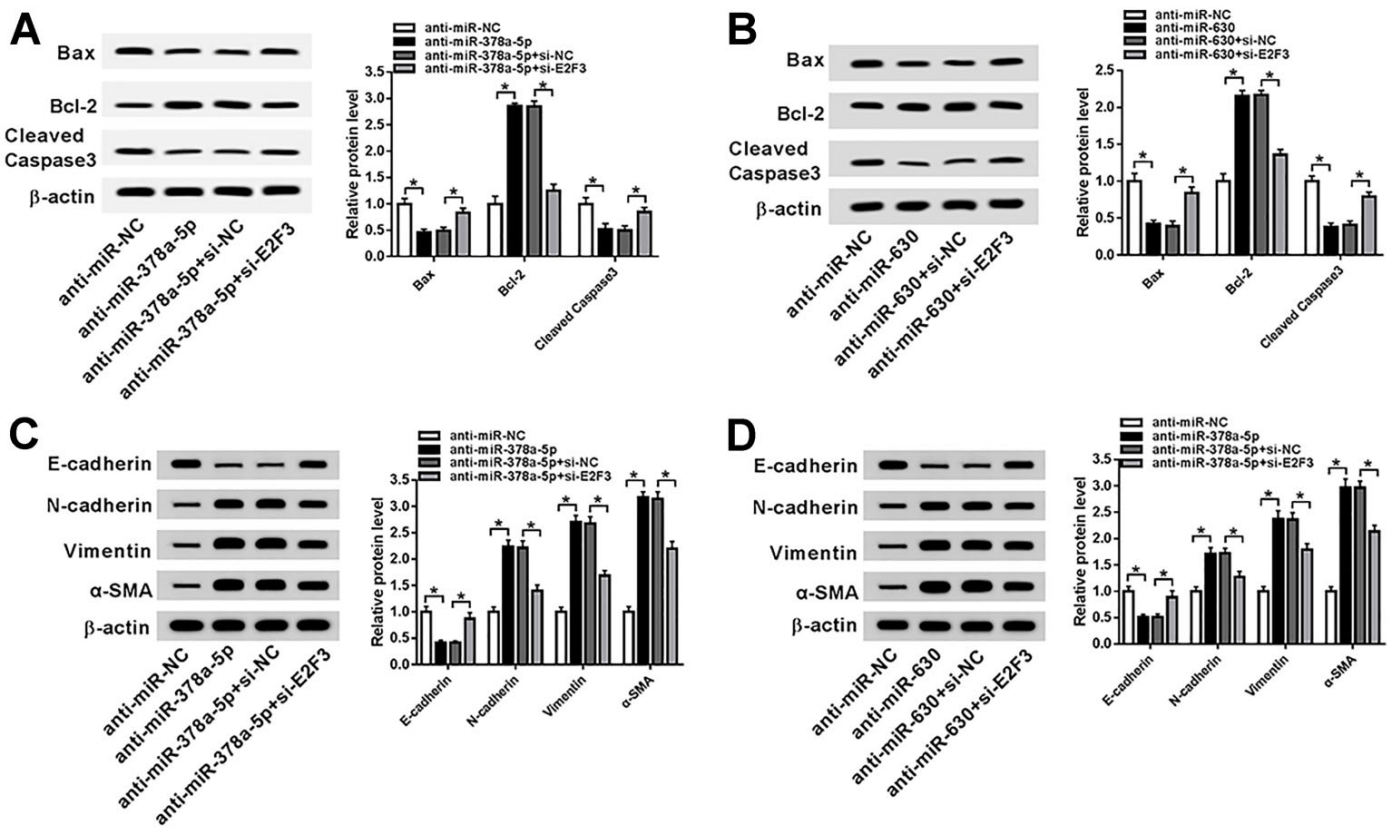
Western blot was used to examine the protein expression levels in transfected SRA01/04 cells of Bax, Bcl-2, and cleaved caspase 3 (**A** and **B**), as well as with E-cadherin, N-cadherin, vimentin, and α-SMA (**C** and **D**). Data are reported as means±SD. *P<0.05 (*t*-test or ANOVA).

As exhibited in Figure 6A and B, the expression of E2F3 mRNA and protein was elevated in SRA01/04 cells transfected with anti-miR-378a-5p and declined in the anti-miR-378a-5p+si-E2F3 transfection group. Consistently, miR-630 inhibitor enhanced the expression of E2F3 mRNA and protein, while E2F3 silencing reversed these effects (Figure 6C and D). In addition, E2F3 silencing rescued miR-378a-5p and miR-630 inhibitor-induced acceleration of cell proliferation (Figure 6E and F) and repression of apoptosis (Figure 6G and H). Also, the decrease of Bax and cleaved caspase-3 as well as the increase of Bcl-2 caused by miR-378a-5p or miR-630 down-regulation were ameliorated following the knockdown of E2F3 (Figure 7A and B). In addition, the intervention of si-E2F3 counteracted the repression of E-cadherin protein expression but promoted N-cadherin, vimentin, and α-SMA protein levels in SRA01/04 cells transfected with anti-miR-378a-5p or anti-miR-630 (Figure 7C and D). Altogether, miR-378a-5p and miR-630 were able to regulate cell progression by interacting with E2F3 in cataract.

## Discussion

Previous studies demonstrated that miR-378a-5p and miR-630 were essential biomarkers of a variety of human diseases. For instance, miR-378a-5p facilitated diosbulbin B-stimulated G2/M cell cycle arrest of hepatocytes by inhibiting CDK1 (29). Also, miR-378a-5p was recognized as a tumor suppressor to reduce cell proliferation and colony formation in colorectal cancer by targeting IGF1R through activating of AKT/ERK pathway (30). However, the role of miR-630 in different cancers is controversial. MiR-630 serves as a tumor promoter in renal cell carcinoma to promote cell migration and invasion and suppress apoptosis (31). On the contrary, miR-630 acts as a tumor inhibitor to block cell proliferation and EMT in gastric cancer by regulation of Wnt/b-catenin pathway (32). Jin et al. (33) also reported that miR-630 hindered cell proliferation, EMT, invasion, and metastasis *in vitro* and *in vivo* in esophageal squamous cell carcinoma. However, the regulatory effects of miR-378a-5p and miR-630 in cataract are largely obscure. In the current study, our experimental results proved that miR-378a-5p and miR-630 reduced cell proliferation and EMT but motivated cell apoptosis in cataract, insinuating the stimulating effects of miR-378a-5p and miR-630 on the progression of cataract.

Based on bioinformatics analysis by Targetscan, both miR-378a-5p and miR-630 could bind to E2F3. Generally, E2F3 is a class of DNA binding proteins regulated by Rb. Hence, E2F3 can induce apoptosis by stimulating DNA damage and activating death-inducing genes (34). Interestingly, E2F3 has been identified as a promising biomarker in many diseases (35,36). For example, enhanced expression of E2F3 was reported to facilitate endothelial cell growth and further accelerate ischemic cardiac repair in ischemic heart disease (37). Abundance of E2F3 contributed to insulin secreting β cell proliferation, providing promising alternative therapy for diabetes (38). In addition, activation of E2F3 was reported to expedite cell proliferation and migration and block apoptosis in bladder cancer by interacting with Rb (39). Conversely, elimination of E2F3 attenuates proliferation, migration, and invasion and accelerates apoptosis of glioma cells (40). Herein, we found that E2F3 overexpression resulted in the enhancement of cell proliferation and EMT, while inhibiting cell apoptosis, suggesting that E2F3 played the repressor gene role in cataract.

We hypothesized that miR-378a-5p and miR-630 could interact with the target gene E2F3 and further regulate cell behavior in cataract. As expected, dual-luciferase reporter assay validated the interaction between E2F3 and miR-378a-5p or miR-630. Besides, miR-378a-5p or miR-630 could negatively regulate the expression of E2F3 mRNA and protein. The rescue experiment showed that E2F3 silencing neutralized the miR-378a-5p or miR-630 inhibitor-mediated promoted effect on cell proliferation but had an inhibitory influence on apoptosis in cataract. Moreover, changed expression of apoptosis-associated proteins (Bax, Bcl-2, and cleaved caspase 3) and EMT-related proteins (E-cadherin, N-cadherin, vimentin, and α-SMA) indicated that miR-378a-5p and miR-630 could stimulate apoptosis and suppress EMT process by targeting E2F3.

In conclusion, we clarified the underlying biological mechanisms of miR-378a-5p/ and miR-630/E2F3 for cataract cell progression. We found that both miR-378a-5p and miR-630 restrained cell proliferation and the EMT process but induced apoptosis of lens epithelial cells via inhibiting E2F3, providing prospective biomarkers for cataract treatment.
